# Multi-omics analysis reveals that low cathepsin S expression aggravates sepsis progression and worse prognosis via inducing monocyte polarization

**DOI:** 10.3389/fcimb.2025.1531125

**Published:** 2025-03-06

**Authors:** Xiao-Ting Luo, Hui-Rong Hu, Zhen-Dong Sun, Li-Hong Zhang, Yan Li

**Affiliations:** Department of Anesthesiology, The Second Affiliated Hospital of Fujian Medical University, Quanzhou, China

**Keywords:** *CTSS*, Mendelian randomization, monocyte, multi-omics analysis, prognosis, sepsis

## Abstract

**Background:**

Monocytes represent a vital cellular subpopulation in the peripheral blood, crucial in the progression of sepsis. Nonetheless, the prognostic role and precise function of monocytes in sepsis are still inadequately understood.

**Methods:**

Single-cell transcriptomic sequencing and bioinformatics analysis were performed on peripheral blood samples from septic patients to identify key molecules in cell subsets. Subsequently, the expression pattern of this molecule was validated through diverse biological experiments, encompassing quantitative RT-PCR, western blotting, and immunofluorescence. Finally, the functionality of this molecule was evaluated using its specific agonist.

**Results:**

A total of 22 monocytes-related biomarkers were identified from single-cell and bulk RNA-seq analyses. Initially, LASSO analysis was performed to derive a prognostic signature composed of 4 key genes, including *CD14*, *CTSS*, *CXCL8* and *THBS1*. Subsequently, mendelian randomization and survival analysis demonstrated that only *CTSS* showed crucially protective role in sepsis development and prognosis. Next, *CTSS* was confirmed to be lower expressed in peripheral monocytes of septic patients. Inflammatory markers (*p* < 0.05) and migration ability of LPS-activated monocytes were significantly reduced after *CTSS* agonist. In addition, *CTSS* agonist decreased the pulmonary tissue monocyte/macrophages infiltration in septic mice.

**Conclusion:**

Monocyte marker *CTSS* represent a promising target for the diagnosis and prognosis evaluation of sepsis and plays a critical role in monocytes activation, tissue inflammatory response and macrophages infiltration. Thus, *CTSS* agonist probably serves as new drug for clinical protection against sepsis.

## Introduction

Sepsis is a major cause of morbidity and mortality for critically ill patients worldwide. It’s estimated that there were 48.9 million of newly diagnosed patients with sepsis in 2017, leading to more than 11 million deaths that already accounted for 20% of the mortality rate around the world (Rudd et al., 2020). Sepsis is commonly involved in multiple organ dysfunction induced by sever systemic inflammation and infection (Markwart et al., 2020). Although the development of bundle strategies has been obtained great advances, severe sepsis is still a leading cause of death (Li et al., 2020; Wang et al., 2021). In the aspect of pathological mechanism, sepsis is involved in the overactivation of innate immune cells, causing uncontrolled inflammation response that results in multiple organ dysfunction in case of severe septicemia (Bosmann and Ward, 2013; Delano and Ward, 2016). Thus, exploring the underlying mechanisms and biomarkers related to sepsis progression and prognosis is necessary.

Monocytes, a crucial kind of peripheral blood mononuclear cells (PBMCs), are the first line of defense against pathogens and are very important to innate and adaptive immune responses by pathogen recognition, antigen presentation, cytokine production, and the expression of costimulatory proteins initiating adaptive immunity (Geissmann et al., 2010). Sepsis promotes the activation of monocyte and influences subset distribution, thus activating lymphocytes and adaptive immune cells, leading to alterations in plasma cytokine levels. It has been demonstrated the functional state of monocytes as a crucial marker ensuring the development and outcomes of sepsis (Li et al., 2020). In addition, monocytes are also reported to play a bidirectional effect on inflammation promotion, resolution and repair, emphasizing the demand to explore the regulatory mechanisms affecting their function (Avendaño-Ortiz et al., 2023). Importantly, elucidating the biological function of monocytes in sepsis probably provides novel therapeutic strategies to intervene their activity.

Phenomenal development in single-cell RNA sequencing (scRNA-seq) technologies greatly promotes the advance in the understanding of the pathogenesis and treatment of sepsis (Wang et al., 2021; Liu et al., 2023). Recently, scRNA-seq elucidates distinct immune cell subpopulations and provides a novel way to define potential diagnostic and prognostic biomarker for sepsis (Li et al., 2022; Wang et al., 2024). In this study, we conducted an integrative analysis of scRNA-seq, Mendelian randomization (MR) and bulk RNA-seq of sepsis to validate monocyte marker genes and determine the key markers with significant prognosis significance. The gene expression omnibus (GEO) samples were applied to further assess the predictive power of the key markers. Additionally, we constructed a clinical nomogram to predicate the 28-days survival probability of sepsis based on the expression of the key markers. Finally, we performed a series of experiments to verify the expression change of the key markers in peripheral blood monocytes from sepsis patients and reveal their function on monocyte activity.

## Materials and methods

### Single-cell transcriptome analysis and transcriptome data acquisition

In this study, we obtained scRNA-seq data from 8 blood samples of sepsis patients from the GEO database (GSE151263 and GSE167363). Firstly, we filtered out clusters with cell counts less than 3, cells with the number of genes mapped less than 300, cells with more than 15% of mitochondrial genes, cells with more than 60% of ribosomal genes and cells with more than 0.5% of erythrocytic genes. Then, data normalization was performed via “NormalizeData” package and the top 40 principal components (PCs) were extracted through principal component analysis (PCA). Subsequently, the Harmony algorithm was used to integrate the data from multiple samples and remove batch effects (Korsunsky et al., 2019). Uniform manifold approximation and projection (UMAP) was utilized for unsupervised clustering and unbiased visualization of cell subpopulations on a two-dimensional map. Cell cluster annotations were based on canonical gene markers. When comparing the differences of gene expression level between a cluster and all other clusters, differential expression analysis was conducted to compare the expression differences of gene between a cluster and all other clusters using the “FindAllMarkers” function. |log2 (fold change) | > 1.5 and P-value< 0.05 were set as the threshold of marker genes for each cluster. Finally, we annotated the cell subpopulations of the different clusters using the “SingleR” package (Aran et al., 2019). We downloaded the RNA-sequencing data and clinical information of blood samples of sepsis patients from GEO database (http://www.ncbi.nlm.nih.gov/geo/).

### Weighted gene co-expression network analysis

The WGCNA package was used to screen genes most involved in monocytes proportion (Langfelder and Horvath, 2008). The overall relevance of all samples in the dataset was ascertained via clustering samples and then outliers was removed. The optimum soft thresholding power β was determined and selected according to the lowest power for which the scale-free topology fit index had a high value. 30 was set as the minimum gene number/module and 3 modules were produced. Eventually, the correlation analysis between modules and traits was performed to explore the most relevant modules for monocytes content.

### Determination and validation of a monocyte-related prognostic signature

First, 90 genes related to monocytes were obtained from single-cell transcriptome analysis and 7749 antiquewhite module genes from WGCNA. Through taking the intersection of the two, we demonstrated 22 candidate monocyte-related genes for sepsis. Subsequently, in order to obtain monocyte-related genes that is involved in prognosis, least absolute shrinkage and selection operator (LASSO) regression analyses were performed.

### eQTL analysis of exposure data

For demonstrating genetic variants involved in gene expression, eQTL analysis was conducted, using transcriptome and genotype data from a variety of cohorts. Summary eQTL data leveraged in the current study were obtained from the GWAS Catalog website (https://gwas.mrcieu.ac.uk/). The “TwoSampleMR” package was then employed to confirm strongly associated SNPs (*p* value<5e ***−*** 8) as instrumental variables (IVs). The threshold of linkage disequilibrium was set at r^2^ < 0.001 and clumping distance = 10,000 kb. According to the filter of “F-test value >10”, the week SNPs were removed.

### Differentially expressed gene screening and functional enrichment analysis

The cathepsin S (*CTSS*) gene was selected based on the aforementioned analysis for further research, and we compared the expression level of signature genes in blood samples of sepsis or the normal control. Subsequently, according to the expression levels of *CTSS*, the genes in GSE dataset were classified into a low expression group and a high expression group. The differentially expressed genes (DEGs) were then screened via the Limma package. The parameters |log2FC|>0.5 and *p* < 0.05 were used as the screening criteria for DEGs. Additionally, volcano plot and heatmap of DEGs were drawn using ggplot2 and pheatmap R packages.

To reveal the potential biological functions of *CTSS*, we used the R package “clusterProfiler” to analyze Gene Ontology (GO) enrichment of DEGs (Yu et al., 2012). GO terms, including biological processes (BPs), with adjusted *p* < 0.05, were considered statistically significant.

### Establishment of the nomogram

In order to explore the clinical factors and the value of *CTSS* for clinical prognosis of patients with sepsis, this study used the R package rms to establish a prediction nomogram that set 1-, 2-, and 3-week OS as the endpoints. Additionally, the calibration curve was utilized to assess the accuracy and resolution of the nomogram. In addition, “survivalROC” R package was used to depict the time-dependent receiver operating characteristic (ROC) curves and the area under curve (AUC). Finally, decision curve analysis (DCA) was also performed to evaluate the net clinical benefit of nomogram on patient prognosis (Petanceska et al., 1996).

### Isolation and culture of PBMCs

We screened sepsis patients according to the following inclusion and exclusion criteria. The inclusion criteria were (1) a suspected or documented infection at the time of admission to the ICU; (2) compliance with the international consensus definition of sepsis (Sepsis-3.0); (3) age ≥ 18 years; (4) enrollment within 24 hours of hospital arrival. The exclusion criteria were (1) age < 18 years; (2) other infections and diseases, such as coronary heart disease, malignant diseases, aseptic inflammation, cerebral infarction, and brain injury. We collected 1ml of peripheral blood samples from healthy volunteers and sepsis patients, which was approved by the Ethics Committee of Second Affiliated Hospital of Fujian Medical University (Approval number: 2024542). After collecting blood samples via venipuncture and mixing it thoroughly with equi-voluminal whole blood and tissue diluent, the mixture was slowly added to a centrifuge tube containing equi-voluminal separation solution (Solarbio, Beijing, China). The centrifuge tube was then centrifuged at 2000 rpm for 30 min at room temperature. We then transferred the second layer of milky white lymphocytes to another centrifuge tube and added 10 ml cell washing solution. After being centrifuged at 1500 rpm for another 10 min, the supernatant was removed and 10 ml cell washing solution was added again. This procedure was repeated three times to isolate monocytes from peripheral blood samples. Once isolated, the monocytes were suspended and cultured in RPMI1640 medium with 10% FBS and 1% P/S.

### Cell culture

The RAW264.7 and THP-1 cell lines were purchased from the Pricella Life Science & Technology Co., Ltd (Wuhan, China). The THP-1 and RAW264.7 cell lines were cultured in 1640 medium with 10% fetal bovine serum (FBS) and 1×penicillin-streptomycin. Cells were routinely cultured in a CO_2_ incubator at 37°C.

### Real-time quantitative PCR

We extracted RNA from cells using TRIzol (Gene Copoeia, MD, USA) and performed the reverse transcription of 1 µg total RNA from each sample to cDNA via the PrimeScript RT reagent Kit (RR047A, Takara, Japan). The reaction conditions were listed below: 37°C for 15 min and 85°C for 5 s. RT-qPCR was then performed on the Bio-Rad Real-Time PCR cycler. The reaction mixture contained TB Green Premix Ex Taq II 5 μL, upstream primer 0.4 μL, downstream primer 0.4 μL, cDNA 2 μL, and RNase Free dH_2_O_2_ 2 μL. The reaction conditions were as follows: 95°C for 30 s, followed by 40 cycles of 95°C for 10 s and 60°C for 30 s. Each well was set up with 3 replicates. The relative expression levels of genes were calculated using the 2^-ΔΔct^ method. The primer sequences were listed in **Supplementary Table S1**.

### Western blotting

After determining the concentration of protein using a BCA protein assay kit (Thermo), equal amounts of proteins (40 µg) in each sample were separated by 12% SDS-PAGE and then transferred to a PVDF membrane (Millipore, Billerica, MA, USA). Subsequently, we blocked the membrane using 5% skimmed milk at room temperature for 1 h and incubated them with the anti-*CTSS* (Signalway, cat. 43650; 1:800) and anti-β-actin (Affinity, cat. AF7018, 1:10000) primary antibodies. Thereafter, the membranes were incubated with the horseradish peroxidase (HRP)-conjugated secondary antibody. Finally, the images were visualized using the ChemiDoc imaging system (Bio-Rad) and blots were quantified via Image J software.

### Chemotaxis assay

Peripheral monocytes (1×10^5^) from sepsis patients or healthy volunteers were added to the upper chamber of the Transwell plate (24-well plate, 5 μm pores; Corning, NY, USA) in 200 μL of serum-free medium. RPMI 1640 medium supplemented with 10% FBS and 1% P/S was added to the lower compartment of the plate. After incubation at 37°C for 24 h, the migrated cells on the lower surface of the membrane were fixed in 10% formalin and then stained with crystal violet. Finally, we selected five random fields of each well to be photographed, and the number of cells were obtained. The experiment was repeated three times independently.

### Animal models of sepsis

Male C57BL/6 mice were maintained in a temperature-controlled environment with a regular 12-hour light and dark cycle. All procedures in this study were approved by the Ethics Committee of Second affiliated hospital of Fujian Medical University (ethical approval number: 2024542). Sepsis model was established via cecal-ligation puncture (CLP) by referencing a surgical procedure as previously described. In brief, the cecum was exteriorized and ligated after anesthesia with 2–4% sevoflurane (inhaled), and then punctured with a 21G needle. After 12 hours post-CLP, mice were euthanized with a pentobarbital overdose (100 mg/kg) and tissue samples were preserved at −80°C for further immunofluorescent staining.

### Statistical analysis

Statistical analysis was conducted using SPSS software (version 26.0; SPSS, Chicago, IL, USA). Data are expressed as mean ± standard deviation (SD). The quantitative data between two groups were compared and analyzed by Wilcoxon test (two-tailed), while a one-way analysis of variance (ANOVA) was used to compare multiple groups, followed by Bonferroni *post hoc* tests. All images were drawn using R version 4.1.3 (https://www.r-project.org/) and its adequate packages. The threshold of statistical significance was set at *p* < 0.05.

## Results

### Identification of monocyte marker genes expression profiles

**Figure 1:**
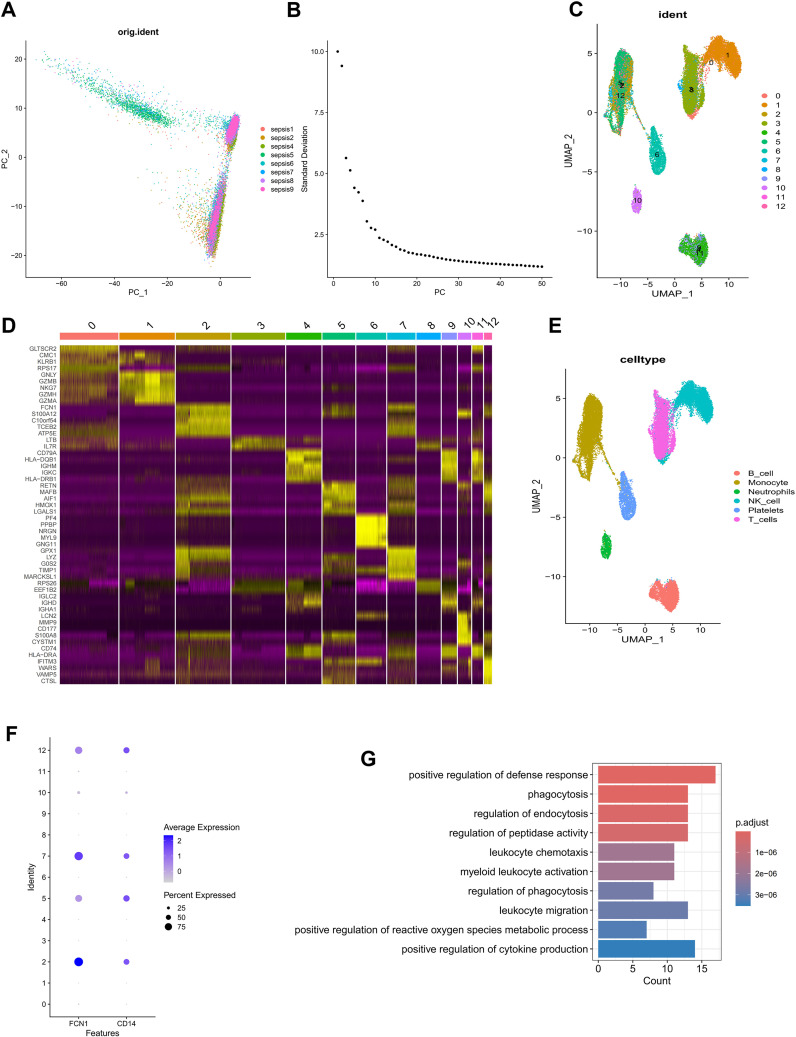
Identification of monocyte marker genes by scRNA-seq analysis. **(A)** PCA was used for dimensionality reduction. **(B)** 40 PCs were selected. **(C)** 13 clusters were visualized based on the UMAP algorithm. **(D)** The heatmap showed the relative expression of genes in 13 clusters. **(E)** Cell subpopulations identified by marker genes. **(F)** The expression level of monocyte marker genes (FCN1 and CD14). **(G)** GO enrichment of monocyte signature genes.

In order to detailedly depict the cellular constitution of blood sample at single-cell resolution and demonstrate the cell markers of monocytes, we reanalyzed the scRNA-seq data of blood samples from 8 sepsis patients. **Supplementary Figure S1A** displayed the depth of sequencing, the range of detected gene numbers, and the percentage of mitochondria, hemoglobin and ribosome content in each sample. After removing low-quality cells through strict quality control, 27069 cells were included in the subsequent analysis. We used the PCA method for dimensionality reduction (**Figure 1A**), and selected 40 PCs for further analysis (**Figure 1B**). Subsequently, Harmony algorithm was used to integrate the data and correct the possible batch effects. We then obtained 13 clusters, which were visualized using the UMAP algorithm (**Figure 1C**). The relative expression of marker genes in cell subpopulations were presented in the heatmap (**Figure 1D**). The “singleR” algorithm was utilized to annotate cell subpopulations and indicated that clusters 2, 5, 7 and 12 were defined as monocyte subpopulations (**Figure 1E**). Due to the special focus on the monocytes in this study, we scrupulously annotated these clusters again using canonical markers (FCN1, CD14) and determined their identity of monocyte subpopulations (**Figure 1F**; **Supplementary Figure S1B**). Finally, we obtained 90 monocyte marker genes of sepsis according to |logFC| >1.5 and *p* value< 0.05 (**Supplementary Table S2**). As expected, the GO enrichment analysis suggested that monocyte gene markers were enriched in biological processes including positive regulation of defense response, phagocytosis and regulation of endocytosis (**Figure 1G**).

### Screening for monocyte-related genes by WGCNA in sepsis

**Figure 2:**
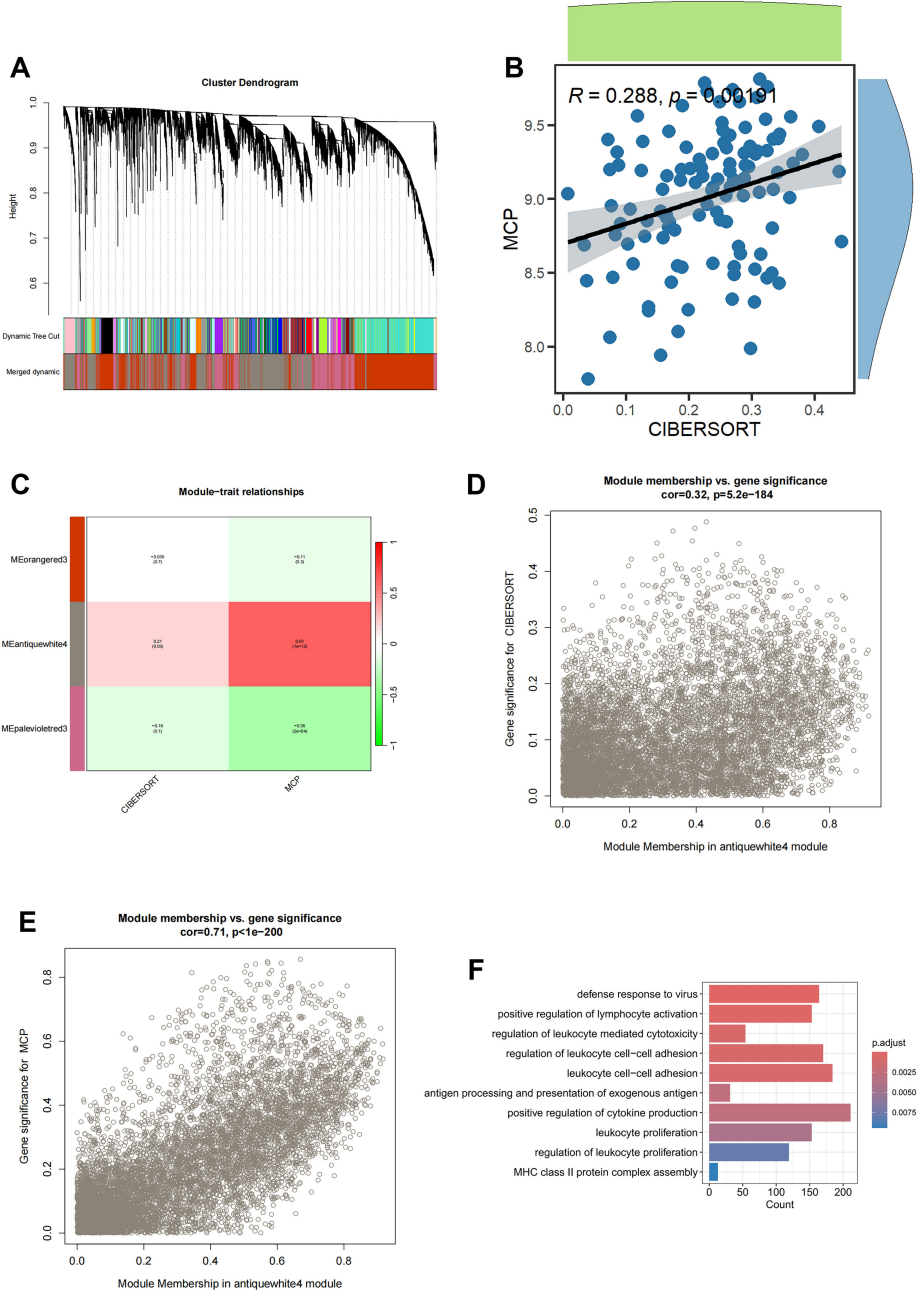
Screening for monocyte-related genes by WGCNA in sepsis. **(A)** The cluster dendrogram constructing the gene modules and module merging. **(B)** Correlation plot of infiltration of monocyte related genes by CIBERSORT and MCPCOUNTER. **(C)** Correlation analysis of modules with traits yielded 3 modules, with the antiquewhite4 module considered to be the most relevant module for monocyte related genes. **(D)** Scatter plot between the antiquewhite4 module and CIBERSORT. **(E)** Scatter plot between the antiquewhite4 module and MCPCOUNTER. **(F)** GO enrichment of antiquewhite4 module genes.

To fully anatomize monocyte-related biomarkers, we performed WGCNA in the bulk RNA-seq dataset of blood sample in sepsis. Initially, after removing the outliers from the blood samples, six were determined as the optimal soft-threshold power and the modules were demonstrated by WGCNA algorithm (**Figure 2A**, **Supplementary Figure S2**). We compared data from two immune infiltration algorithms, including MCPCOUNTER and CIBERSORT, as we expected, there was excellent congruity on the percentage of monocytes in both data (R=0.288, P=0.002; **Figure 2B**). In order to precisely determine the key modules of WGCNA involved in monocytes, we separately calculated the correlation between the two types of immune infiltration data and the modules. As displayed in **Figure 2C**, the antiquewhite4 module showed the highest correlation to the deconvolution result. Thus, we demonstrated the gene in the antiquewhite4 module as a potential biomarker for monocytes in sepsis (**Figures 2D, E**; **Supplementary Table S3**). In addition, amazingly, the results from the GO enrichment analysis of the antiquewhite4 module genes were consistent with the enrichment analysis of the monocytes marker genes demonstrated by scRNA-seq data (**Figure 2F**). In conclusion, our analysis identified an obvious correlation between the antiquewhite4 module and monocytes in sepsis via WGCNA.

### Demonstration of a monocyte-related prognostic marker in sepsis

**Figure 3:**
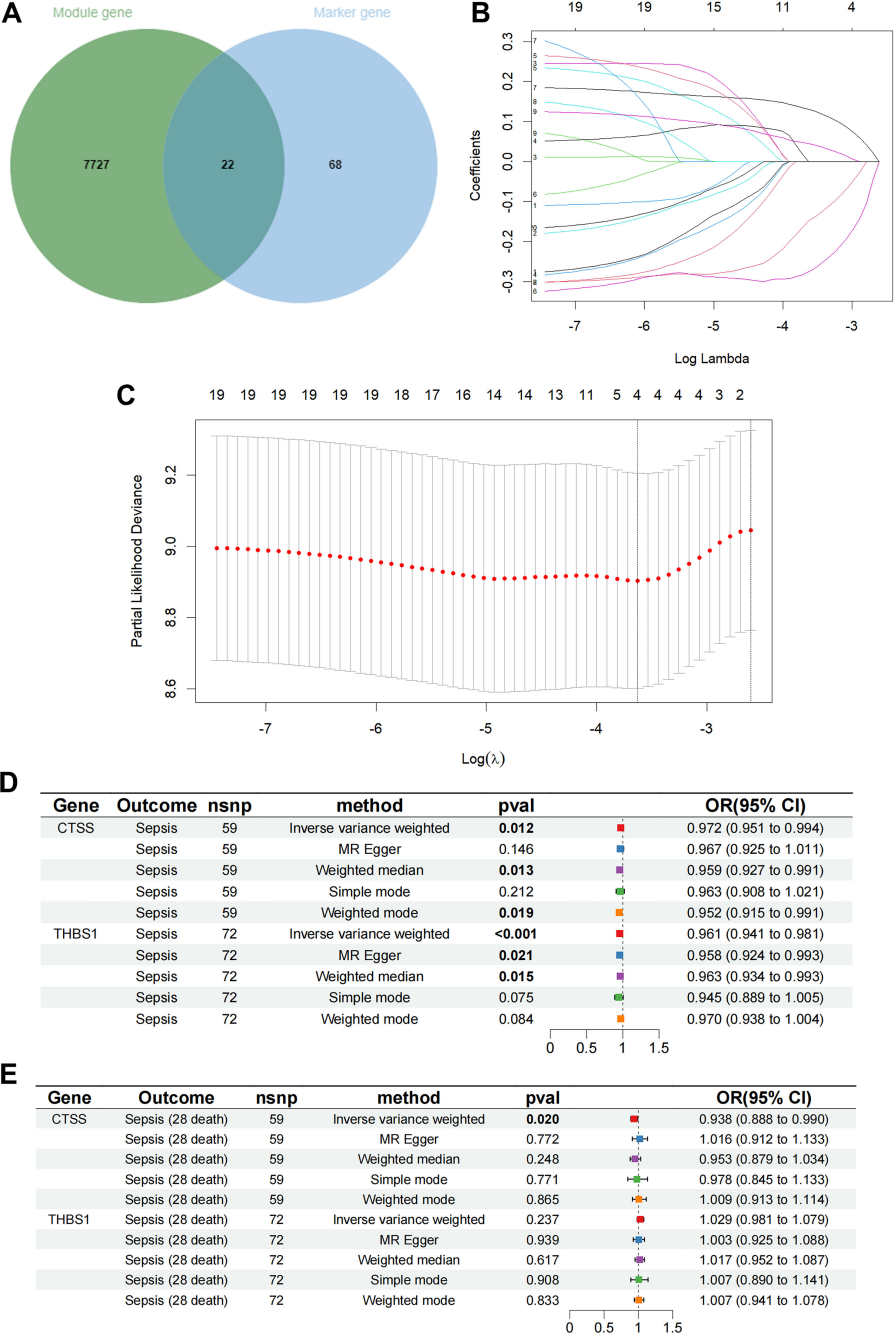
Determination of key monocyte-related genes. **(A)** The Venn graph of the monocyte signature genes and antiquewhite4 module genes. **(B)** Coefficient profiles in the LASSO regression model. **(C)** Cross-validation for tuning parameter selection in the LASSO regression. **(D)** The causal relationship of key genes and sepsis risk. **(E)** The causal relationship of key genes and 28 days mortality.

**Figure 4:**
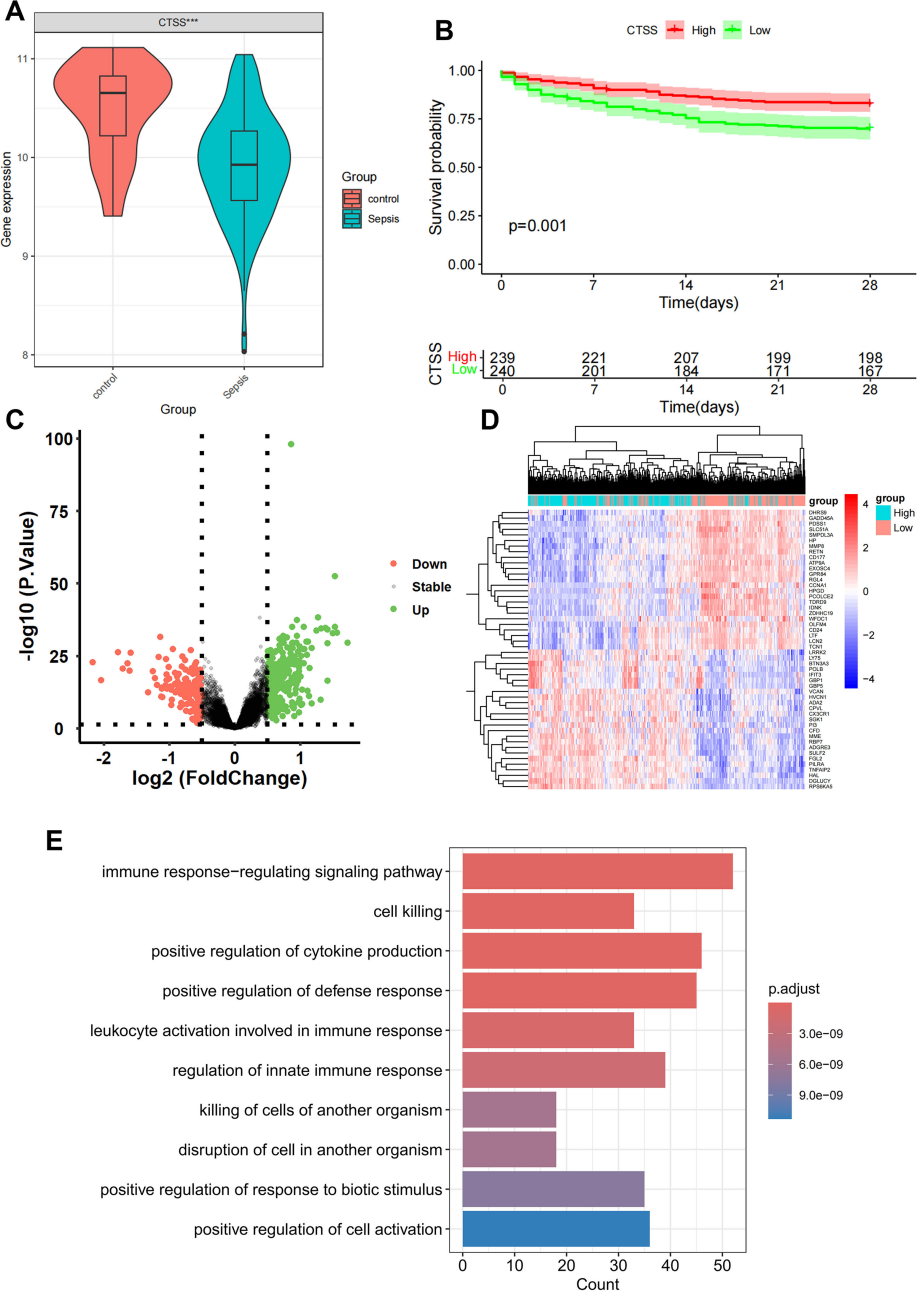
Differential and enrichment analysis of CTSS grouping. **(A)** Boxplot showing the expression difference of CTSS in control and sepsis groups. **(B)** K-M plot showing the prognostic significance of CTSS in sepsis. **(C)** Differential analysis volcano graph for CTSS expression. **(D)** The differential analysis heat map of CTSS high and low expression groups. **(E)** Differentially expressed genes (DEGs) in Gene Ontology (GO) analysis.

By intersecting the 90 monocyte marker genes with the 7749 antiquewhite4 module genes for fetching, 22 candidate monocyte-related genes were obtained (**Figure 3A**). Initially, LASSO analysis was performed to derive a prognostic signature composed of 4 key monocyte-related marker genes (MRMGs), including cluster of differentiation 14 (*CD14*), *CTSS*, C-X-C motif chemokine ligand 8 (*CXCL8*) and Thrombospondin 1 (*THBS1*) (**Figures 3B, C**; **Supplementary Table S4**). Subsequently, we investigated the causal effect of key MRMGs expression on sepsis risk and mortality using their e-QTL data from GWAS Catalog website (https://gwas.mrcieu.ac.uk/) via MR analysis. **Figures 3D, E** displayed that only *CTSS* exhibited protective causality with sepsis risk and mortality. Thus, we further examined the expression of *CTSS* in the GSE131761 dataset. We discovered that the expression of *CTSS* was significantly downregulated in sepsis patients than that in control healthy (**Figure 4A**, *p*<0.001). Furthermore, we utilized the survival data of sepsis patients in GSE65682 dataset to further explore the prognostic significance of *CTSS* (**Figure 4B**). We found that patients in the low group of *CTSS* expression displayed obviously worse prognosis when compared with high group. This series of analysis demonstrated the crucially protective role of *CTSS* in sepsis development and prognosis, promoting it to be selected for subsequent analysis.

### Difference analysis and enrichment analysis of *CTSS* grouping

Patients with sepsis in GSE65682 dataset was classified into a low-expression group and a high-expression cohort according to the median value of *CTSS*, with *p* value < 0.05 and |log2FC| >0.5; 161 low-expressed genes and 354 up-expressed genes were obtained (**Figure 4C**). The heat map only showed the top 50 DEGs in |logFC| order (**Figure 4D**). In order to further study the functional effects of different *CTSS* expressions, we first analyzed the 515 DEGs of GSE65682 by GO enrichment analysis. The top five groups functional effects of BP included immune response-regulating signaling pathway, cell killing, positive regulation of cytokine production, positive regulation of defense response, and leukocyte activation involved in immune response (**Figure 4E**). The outcomes of the GO enrichment analysis of DEGs were also similar with the enrichment analysis of the monocyte marker genes and the antiquewhite4 module genes.

### Analyses of clinicopathological characteristics and the establishment of nomogram based on *CTSS* expression

**Figure 5:**
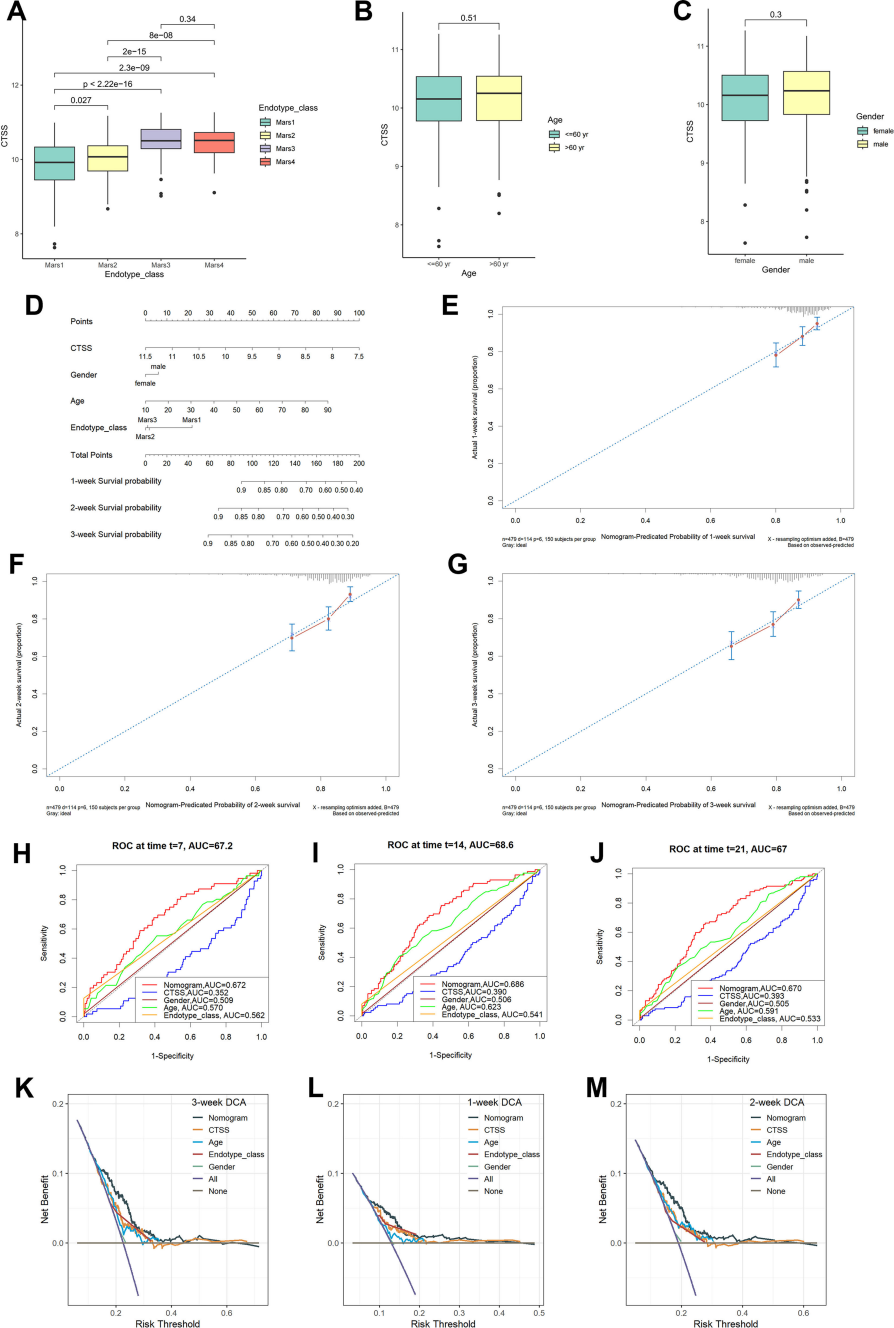
Clinical correlational analysis. **(A)** Expression level of CTSS in different endotypes. **(B)** Expression level of CTSS in different age groups. **(C)** Expression level of CTSS in different sex groups. **(D)** Nomogram plot. **(E–G)** 7-day, 14-day, 21-day survival rate calibration curve. **(H-J)** Time-dependent ROC curve analysis of the nomograms compared for 1-, 2-, and 3-week OS, respective. **(K-M)** The DCA curves of the nomograms compared for 1-, 2-, and 3-week OS, respective.

Besides the obvious survival outcomes found between populations with high/low-expression of *CTSS*, distinct variances were also seen in their clinicopathologic characteristics. An incremental decrease in the expression level of *CTSS* was observed with declining endotype class, indicating a potential association between the expression level and the progression of sepsis (**Figure 5A**). However, the expression level of *CTSS* did not display significant difference in different gender and ages (**Figures 5B, C**). Subsequently, we established a nomogram by integrating clinicopathologic factors and *CTSS* to predict 1-, 2-, and 3-week survival probabilities of sepsis patients, respectively (**Figure 5D**). Calibration plots demonstrated the high consistence between the observed values and the predicted values (**Figures 5E–G**). Additionally, The AUCs showed the nomogram provided more clinical net benefit in forecasting prognosis at 1-, 2-, and 3-weeks (**Figures 5H–J**). DCA showed that the nomogram also held the optimum clinical net benefit for 1-, 2-, and 3-weeks survival (**Figures 5K–M**). These findings indicated that the nomogram based on the expression level of *CTSS* could be served as an effective way to forecast prognosis of sepsis patients in clinical practice.

### Identification of *CTSS* expression trend via *in vitro* and *in vivo* experiments

**Figure 6:**
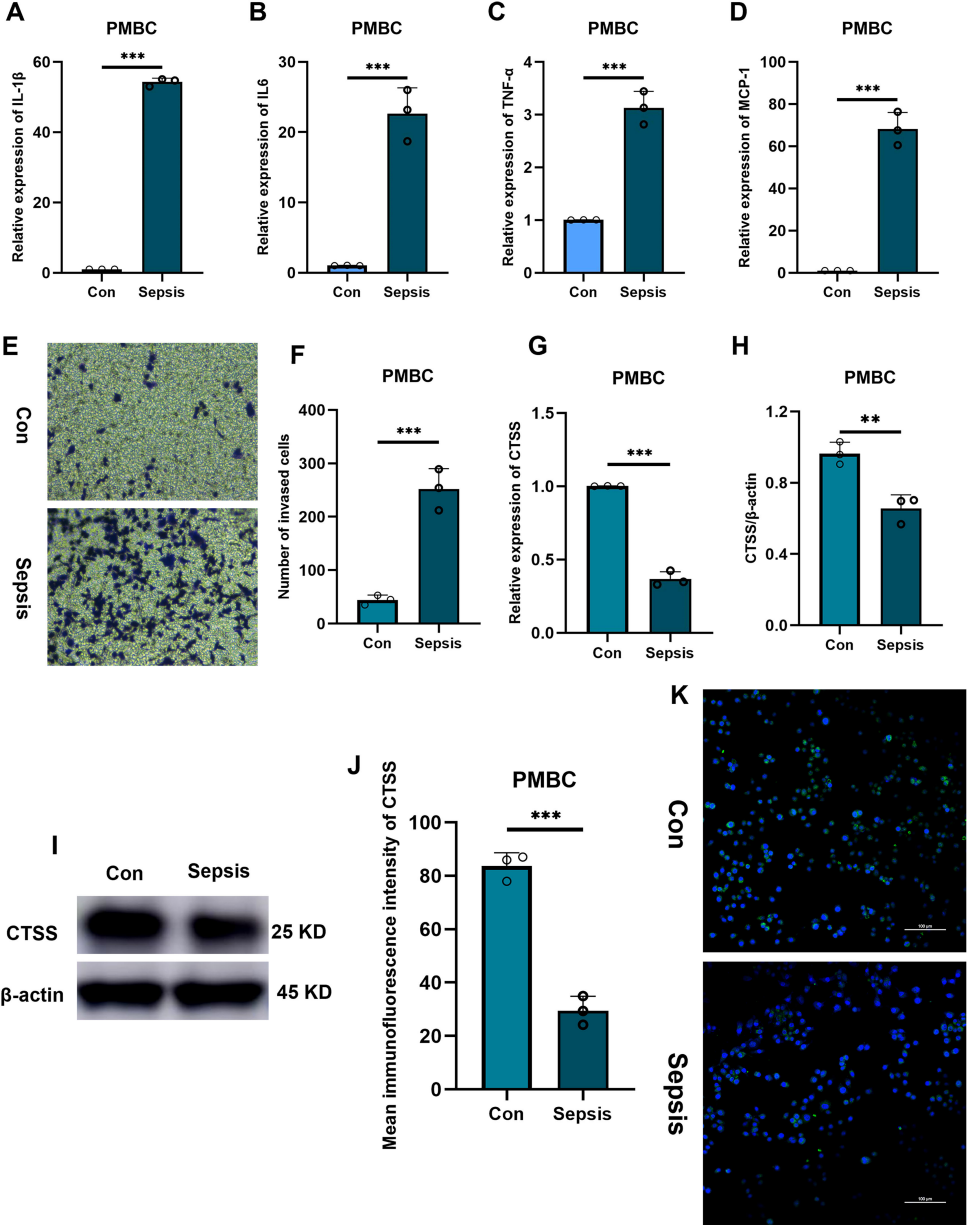
The verification of CTSS expression in PBMC. **(A-D)** RT-qPCR analysis of cytokine expression. **(E, F)** Transwell assay evaluating the chemotactic ability of monocytes from healthy and septic populations. **(G)** RT-qPCR analysis of CTSS expression. **(H, I)** Western blot analysis of CTSS expression. **(J, K)** Immunofluorescence analysis of CTSS expression. **p < 0.01, ***p < 0.001.

**Figure 7:**
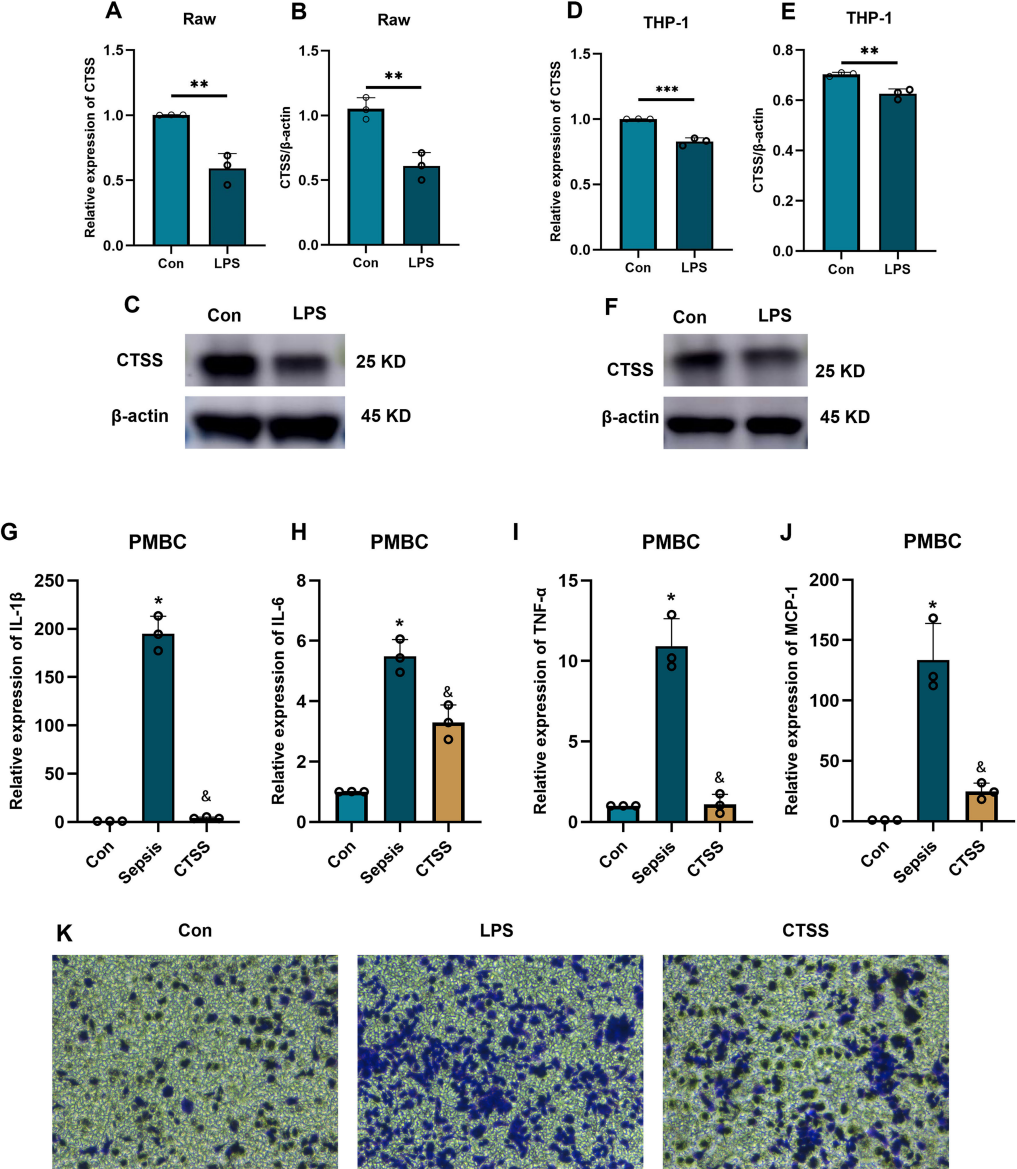
The effect of CTSS on monocyte function and activity. **(A-F)** The verification of CTSS expression in human monocyte/macrophage lines. **(A-C)** RT-qPCR and western blot analysis of CTSS expression in Raw macrophages. **(D-F)** RT-qPCR and western blot analysis of CTSS expression in THP-1 cells. **(G-J)** RT-qPCR analysis of cytokine expression. **(K)** Transwell assay evaluating the chemotactic ability of monocytes. *p < 0.05, **p < 0.01, ***p < 0.001 compared with Con group; &, compared with LPS group.

Aforementioned analysis demonstrated the important role and prognostic significance of monocyte-related marker *CTSS* in sepsis. Interestingly, activation of monocytes is one of main characteristics of sepsis. Thus, we questioned whether sepsis-induced *CTSS* downregulation is involved in activating monocytes. Monocytes were firstly isolated from the peripheral blood of healthy individuals and septic patients using commercial kit. qRT-PCR analysis demonstrated obviously lower expression levels of activated monocyte-related markers (*MCP-1, TNF-α, IL-6, IL-1β*) mRNA in the peripheral monocytes of septic patients, compared to monocytes from healthy individuals (**Figures 6A–D**) (*p* < 0.05). The chemotactic ability of monocytes was obviously stronger in septic patients than that in healthy control (**Figures 6E, F**) (*p* < 0.05). qRT-PCR, western blot and immunofluorescence analysis verified the lower expression levels of the *CTSS* mRNA and protein in the peripheral monocytes of septic patients (**Figures 6G–K**) (*p* < 0.05). The expression level of *CTSS* was subsequently investigated in THP-1 and RAW264.7 cells following LPS stimulation. The findings indicated a significant decrease in the mRNA and protein levels of *CTSS* in both cell lines upon LPS treatment (**Figures 7A–F**) (*p* < 0.05).

### *CTSS* agonist inhibits the activation and chemotaxis of monocytes in an *in vitro* model of sepsis

Following the administration of LPS, we investigated the role of *CTSS* in monocyte activation during sepsis by treating monocytes obtained from healthy individuals with a *CTSS* agonist. The qRT-PCR results (**Figures 7G–J**) showed a significant increase in the expression levels of activated monocyte-related markers (*MCP-1, TNF-α, IL-6, IL-1β*) mRNA in the LPS group compared to the control group. In contrast, the *CTSS* agonist group exhibited a significant decrease in the expression levels of these marker genes compared to the LPS group (*p* < 0.05). Migration analysis (**Figure 7K**) revealed an increase in the number of invaded cells in the LPS group compared to the control group. Conversely, the *CTSS* agonist group exhibited a decrease in the number of invaded cells compared to the LPS group.

### *CTSS* agonist improves the tissue inflammatory response and macrophages infiltration in septic mice

**Figure 8:**
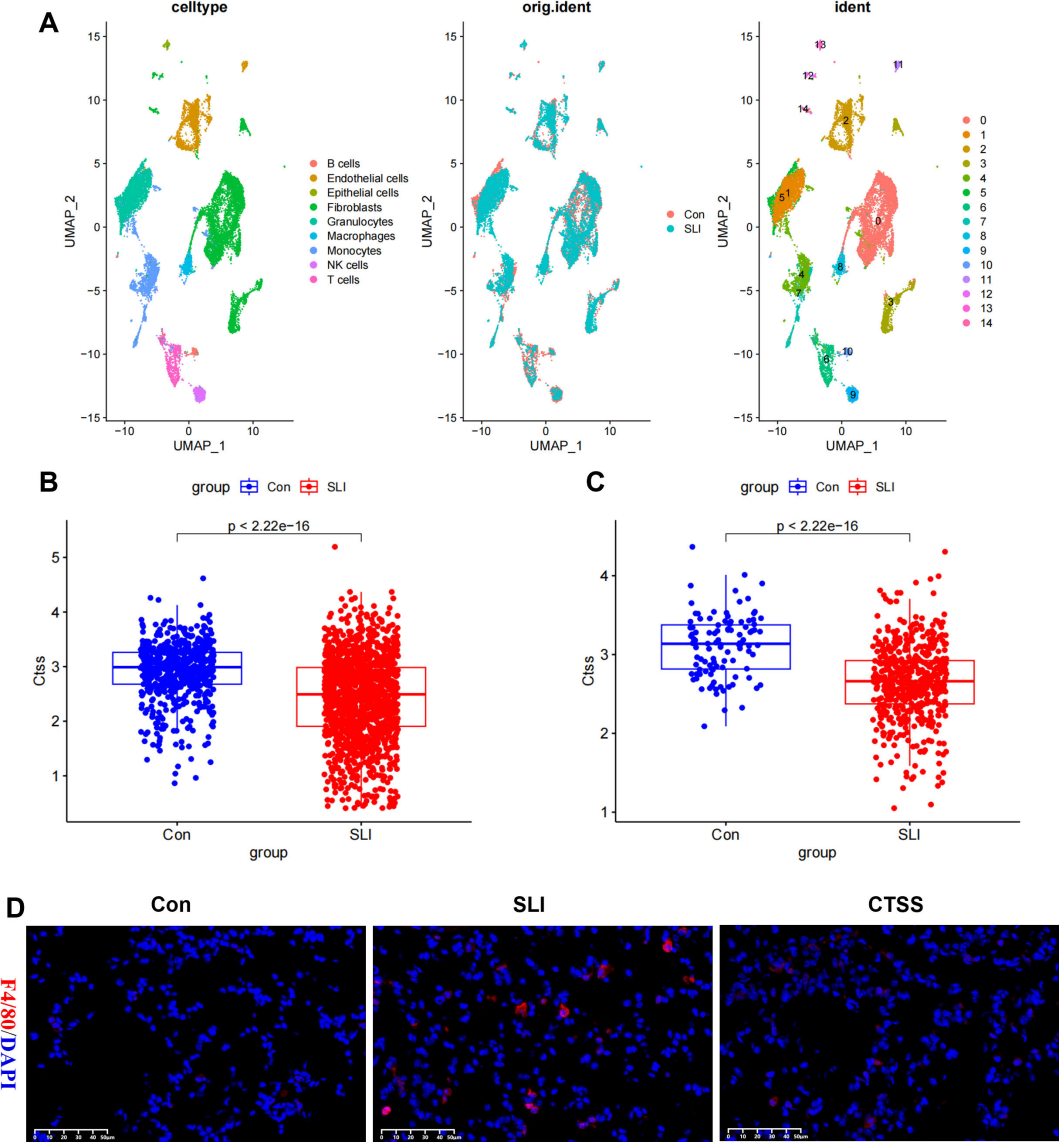
The effect of CTSS on monocyte/macrophage filtration in lung tissue. **(A)** Cell clusters were visualized based on the UMAP algorithm. **(B)** Boxplot showing the expression difference of CTSS in mice lung monocytes in control and sepsis lung injury (SLI) groups. **(C)** Boxplot showing the expression difference of CTSS in mice lung macrophages in control and SLI groups. **(D)** Immunofluorescence staining of the macrophage markers F4/80 (red) and DAPI (blue) in lung sections.

Tissue inflammation is one of major characteristic of sepsis. Thus, we mined the GSE207651 dataset profiling the single-cell immune landscape throughout lung sepsis to understand the impact of *CTSS* on the tissue inflammatory responses of septic mice. After data filtration, integration and removal of potential batch effects, we annotated each cell subpopulation as epithelial cells, T cells, B cells, endothelial cells, fibroblasts, granulocytes, macrophages, monocytes, and NK cells (**Figure 8A**). Thereafter, with a special focus on the *CTSS*, we observed monocytes and macrophages showed significant decrease of this gene in septic lung injury, which was believed to play a role in the function of monocytes/macrophages (**Figures 8B, C**). Subsequently, to further elucidate the possible role of *CTSS* in monocytes/macrophages during sepsis, we assessed the infiltration of macrophages into the septic and normal lungs of mice. The results showed that the number of infiltrated macrophages in the lungs of septic mice were higher than that in the lungs of control mice, as evidenced by the higher F4/80 fluorescence intensity in septic lungs. However, *CTSS* agonist effectively decreased the number of infiltrated macrophages in the lungs of septic mice (**Figure 8D**).

## Discussion

Recently, bioinformatic techniques, including tools and software, have obtained fast and great advancement, promoting public databases to be an excellent resource for decoding the pathophysiology of sepsis. The dysfunction of inflammatory response exists throughout the whole process of sepsis. Monocytes exert an important role in the pathogen clearance and immune regulation. After being activated, monocytes also expressed and released a series of cytokines, contributing to the form of cytokine storm and the activation of innate immune system (Hotchkiss et al., 2013; Nedeva, 2021). However, exploring the mechanism by which monocytes are activated during sepsis remains a great challenge. scRNA-seq technology is a useful tool for investigating cell heterogeneity and different cell subpopulations, which is crucial for founding underlying therapeutic targets (Tu et al., 2023). In the present study, we employed scRNA-seq analysis to excavate monocyte marker genes in sepsis and then found optimal prognostic marker using transcriptomic and MR analysis. Subsequently, we constructed a prognostic nomogram model based on the prognostic marker and clinicopathologic feature of sepsis patients. Finally, the expression and function of the prognostic marker in monocytes were further investigated via a series of experiments.

Bulk RNA-seq technique examines difference between tissue samples at the transcriptomic level. Furthermore, single-cell RNA-seq elucidates variations within tissue samples at the individual cell level (Tu et al., 2023). In contrast, the MR analyses used single nucleotide polymorphisms (SNPs) as instrumental variables (IVs) to evaluate the causal association between exposure and outcome (Emdin et al., 2017). The combination of these three methods provides a multi-omics exploration in the reveal of profound biological insights for sepsis risk and prognosis. In the current study, we re-analyzed single cell sequencing data with 8 blood samples from sepsis patients and distinctly demonstrated monocyte subpopulations, defining 90 monocyte markers. Subsequently, we employed WGCNA to explore the biological markers of monocyte in bulk RNA-seq. In order to make sure the robustness of the marker genes, two bioinformatics methodologies were used to quantify the proportion of monocytes in blood of sepsis patients. Module correlation analysis demonstrated a strong association between the antiquewhite4 module and both two methods, thus confirming the antiquewhite4 module as a marker for monocytes in bulk RNA-seq. Next, we obtained 22 genes involved in monocytes by intersecting the marker genes from scRNA-seq with the monocyte module genes in bulk RNA-seq. LASSO regression analyses was then performed to screen four potential genes with prognostic relevance, including two risk genes (*CXCL8* and *THBS1*) and two protective genes (*CD14* and *CTSS*). MR analysis was employed to further evaluate the casual association of these four marker genes with sepsis risk and prognosis. We found that only *CTSS* showed stable predicative value for sepsis prognosis, which was also demonstrated by Kaplan-Meier analysis.

*CTSS* (cathepsin S) is a cysteine protease primarily expressed in phagocytic cells, including monocytes, lacrimal gland acinar cells, antigen-presenting cells, and macrophages (Petanceska et al., 1996; Wendt et al., 2008; Li et al., 2010). Amongst the fifteen human cathepsins, *CTSS* shows unique distinguishing characteristics, including relatively restricted expression and the maintenance of catalytic ability at neutral/mildly alkaline pH. Recently, the diagnostic value of *CTSS* has been increasingly revealed in some diseases. For example, Ren et al. indicated that increase in serum level of *CTSS* was involved in the progression of albuminuria and reduced renal function in patients with type 2 diabetes mellitus (Ren et al., 2023). Zuo et al. found that increased serum levels of *CTSS* efficiently predicted the occurrence of liver fibrosis, even at an early stage (Zuo et al., 2023). However, no study has evaluated the correlation between *CTSS* and the risk of sepsis. In this study, we demonstrated that monocyte marker gene *CTSS* is a protective factor for sepsis risk and prognosis.

On the other hand, *CTSS* also involves a variety of intracellular and extracellular processes. By the combination of a quantitative proteomic analysis of clinical fibrotic liver samples with *in vivo* experiment, Zuo et al. (2023) demonstrated that macrophage-derived *CTSS* is a central protein for extracellular matrix remodeling and can promote liver fibrogenesis by facilitating the release of endostatin peptide via cleaving collagen 18A1 at its C-terminus. Macrophage-derived *CTSS* was involved in hydrolysis of extracellular matrix components (including laminin, elastin, and collagen), finally leading to vascular damage (Figueiredo et al., 2015). Additionally, monocyte/macrophage-secreted *CTSS* has been demonstrated to facilitate the activation of protease-activated receptor-2 on glomerular endothelial cells, contributing to inflammation, endothelial damage, albumin leakage, and glomerulosclerosis (Kumar Vr et al., 2016). Importantly, a recent study identified that monocytes with high cathepsin S expression aggravated cerebral ischemia–reperfusion injury via promoting blood-brain barrier destruction through junctional protein cleavage (Xie et al., 2023). These findings highlighted the deleterious effect of *CTSS* in the progression of disease. Conversely, Oshima et al. found that M2 macrophage-derived *CTSS* promotes peripheral nerve regeneration via activating Schwann cells via Ephrin-B2 shedding from fibroblasts (Oshima et al., 2023). Similarly, in this study, we also demonstrated that sepsis induced the downregulation of *CTSS* in monocytes and *CTSS* agonist administration effectively inhibited the activation of peripheral monocytes, improved the tissue inflammatory response and macrophages infiltration. These results further demonstrated the protective effect of *CTSS* on sepsis.

There were some limitations in this study. The first is the demographic diversity and small sample size, which possibly influencing the generalizability of the outcomes. Although we used *in vitro* and *in vivo* models to perform further validation, these basic models cannot totally reflect the intricate physiological states and immune environment in humans. In addition, some uncontrollable variables, including lifestyle habits and individual differences, probably also impacted the results. Future efforts should devote to demonstrating the conclusions of the present study via extending sample sizes and performing multi-center clinical trials. Finally, this study did not explore the potential molecular mechanisms of *CTSS* regulation.

In summary, through integrating single-cell sequencing, bulk RNA-seq, MR analysis and experimental validation, we found that monocyte marker *CTSS* represent a promising target for the diagnosis and prognosis evaluation of sepsis and plays a critical role in monocytes activation, tissue inflammatory response and macrophages infiltration. Thus, further exploration into the specific role of *CTSS* in monocytes and its downstream crosstalk with other molecular pathways to reveal its general role in monocyte activities, will be a critical direction of future research. These findings will provide the translational medical research with novel ideas and methods to improve the therapeutic strategies, finally accomplishing the target of personalized medicine.

## Data Availability

The original contributions presented in the study are included in the article/**Supplementary Material**. Further inquiries can be directed to the corresponding author.

## Supplementary material

Supplementary Figure 1
**(A)** Quality control of scRNA-seq data from blood samples of patients with sepsis. **(B)** Feature plots of monocyte marker genes (FCN1 and CD14).

Supplementary Figure 2 Network topology analysis under various soft-threshold powers. Left: The x-axis represents the soft-threshold power. The y-axis represents the fit index of the scale-free topology model. Right: The x-axis represents the soft-threshold power. The y-axis reflects the average connectivity (degree).
