# Supplementary figures and images for: Multi-omics analysis reveals that low cathepsin S expression aggravates sepsis progression and worse prognosis via inducing monocyte polarization
Source: Front Cell Infect Microbiol. 2025 Mar 6;15:1531125. doi: 10.3389/fcimb.2025.1531125 (PMC11922721; doi:10.3389/fcimb.2025.1531125)

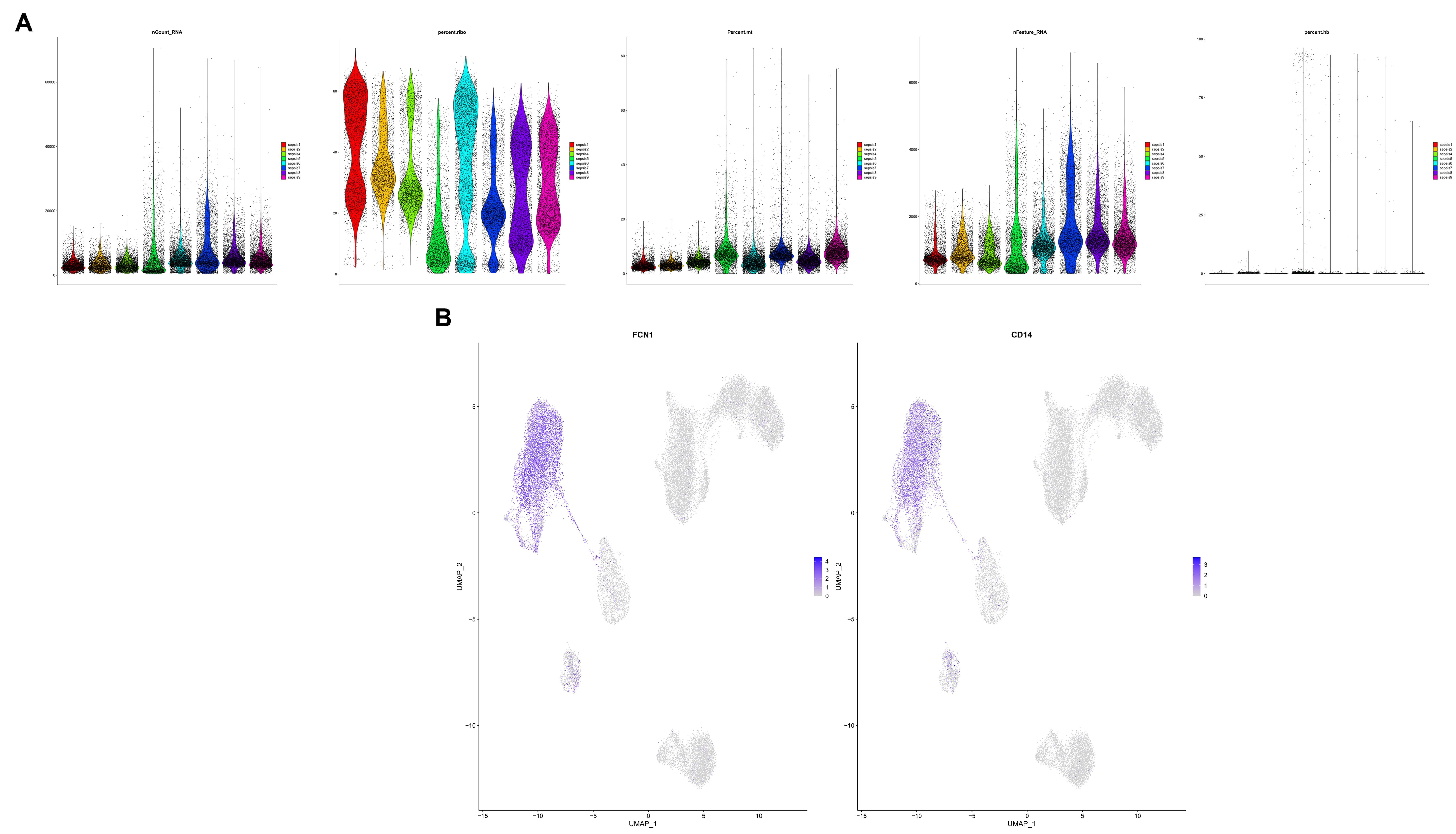

Supplement: Supplementary Figure 1 — (A) Quality control of scRNA-seq data from blood samples of patients with sepsis. (B) Feature plots of monocyte marker genes (FCN1 and CD14). [file Image1.jpeg]

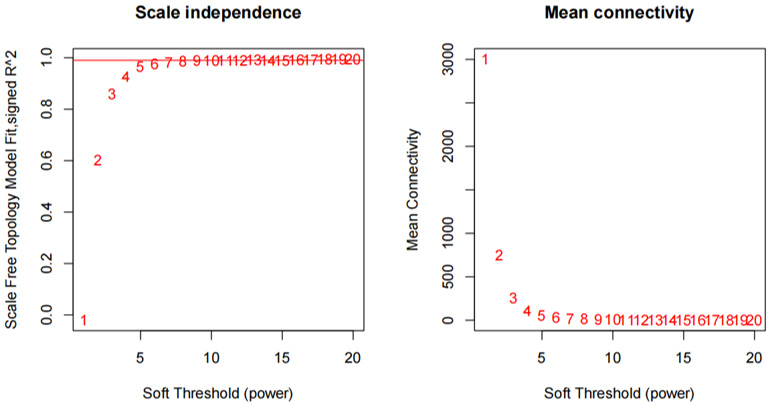

Supplement: Supplementary Figure 2 — Network topology analysis under various soft-threshold powers. Left: The x-axis represents the soft-threshold power. The y-axis represents the fit index of the scale-free topology model. Right: The x-axis represents the soft-threshold power. The y-axis reflects the average connectivity (degree). [file Image2.jpeg]
